# Anatomical traits explain drought response of seedlings from wet tropical forests

**DOI:** 10.1002/ece3.70155

**Published:** 2024-09-01

**Authors:** Rishiddh Jhaveri, Lakshmipriya Cannanbilla, K. S. Arpitha Bhat, Mahesh Sankaran, Meghna Krishnadas

**Affiliations:** ^1^ CSIR – Centre for Cellular and Molecular Biology Hyderabad India; ^2^ Academy of Scientific and Innovative Research (AcSIR) Ghaziabad India; ^3^ Chair of Plant Ecology University of Bayreuth Bayreuth Germany; ^4^ Department of Life Science Bangalore University Bangalore India; ^5^ Ashoka Trust for Research in Ecology and the Environment (ATREE) Bangalore India; ^6^ National Centre for Biological Sciences, TIFR Bangalore India

**Keywords:** above‐and‐below ground plant traits, greenhouse experiment, seasonality affiliation, seedling drought response, tropical tree species

## Abstract

Water availability regulates plant community dynamics but the drought response of seedlings remains poorly known, despite their vulnerability, especially for the Asian tropics. In particular, discerning how functional traits of seedlings mediate drought response can aid generalizable predictions of tree responses to global environmental change. We assessed interspecific variation in drought response explained by above‐ and below‐ground seedling traits. We conducted a dry‐down experiment in the greenhouse using 16 tree species from the humid forests of Western Ghats in southern India, chosen to represent differences in affinity to conditions of high and low seasonal drought (seasonality affiliation). We compared survival, growth, and photosynthetic performance under drought and well‐watered conditions and assessed the extent to which species' responses were explained by seasonality affiliation and 12 traits of root, stem and leaf. We found that the species from seasonally dry forest reduced photosynthetic rate in drought compared with well‐watered conditions, but seasonality affiliation did not explain differences in growth and survival. Performance in drought vs well‐watered conditions were best explained by anatomical traits of xylem, veins and stomata. Species with larger xylem reduced their growth and photosynthesis to tolerate desiccation. In drought, species with smaller stomata correlated with lower survival even though photosynthetic activity decreased by a larger extent with larger stomata. Overall, anatomical traits of xylem and stomata, directly related to water transport and gas‐exchange, played a more prominent role than commonly used traits (e.g., specific leaf area, leaf dry matter content) in explaining species response to drought, and may offer a good proxy for physiological traits related to drought tolerance of seedlings.

## Graphical abstract

Greenhouse experiment investigating the drought response of seedlings from the Asian tropics, focusing on 16 tree species from the Western Ghats, a biodiversity hotspot in India. Assessing survival, growth, and photosynthetic performance under experimental drought treatment, the study reveals that anatomical traits of xylem, veins, and stomata play a more critical role than commonly used soft traits (e.g., specific leaf area, wood density) in explaining species responses to drought. Species found in seasonally dry forests reduce photosynthesis under drought, possibly a drought stress tolerating mechanism. These findings offer insights into predicting tree responses and community dynamics to global environmental change.

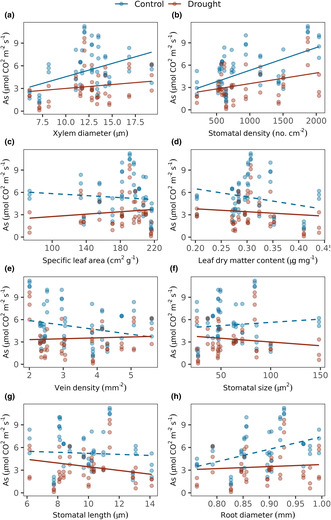

## 1 INTRODUCTION

Water availability drives the distribution and performance of plant species, even within relatively wet biomes (Comita & Engelbrecht, 2009; Engelbrecht et al., 2007; Esquivel‐Muelbert et al., 2019; Gopal et al., 2023; Krishnadas et al., 2021). Quantifying plant response to water stress is imperative given that global environmental change will alter patterns of precipitation and seasonal drought (Hajek & Knapp, 2022; Mishra et al., 2020). For closed canopy forests, much attention has focused on the response of mature trees to drought (Anderegg et al., 2015; Hartmann et al., 2018; Rowland et al., 2015; Suresh et al., 2010), but less is known about seedlings. Seedlings can be highly vulnerable to drought because their shallow roots do not allow access to deeper water layers during dry periods. Moreover, the light‐limited forest understory may impose carbon starvation during drought (Amissah et al., 2015; Slot & Poorter, 2007) such that even short dry spells can result in substantial seedling mortality (Engelbrecht et al., 2007). Seedling responses to water availability can therefore affect regeneration and persistence of species in a changing climate and shape community structure of later life‐stages (Comita & Engelbrecht, 2009, 2014).

Interspecific variation in drought response can be mediated by species' traits—anatomical, morphological and physiological characteristics associated with differences in strategies of resource use (Poorter & Markesteijn, 2008; Slot & Poorter, 2007). For example, wood density and leaf mass per area (LMA) are correlated positively with drought tolerance (Muscarella et al., 2016; O'Brien et al., 2017), but may not play a direct function in the plant's water use efficiency or transport, whereas variation in anatomical features of stomata and xylem are functionally related to water transport and use of water and help regulate water balance and response to dry conditions. Stomatal and xylem traits may thus serve as good proxies for hydraulic function (Bartlett et al., 2016) but are not well‐characterized for tropical tree seedlings, especially in terms of seedling response to drought. In addition, the importance of roots in accessing ground water is commonly understood, but the role of root traits in mediating species' drought responses remains poorly explored. Root traits can be a key component of plant functional strategies and whole‐plant response to drought stress (Freschet et al., 2021; Laughlin et al., 2021; Weemstra et al., 2023), which may be logistically difficult to study for mature trees but convenient for seedlings.

Evergreen species may have evolved to withstand seasonal droughts either by avoiding or tolerating desiccation, with functionally distinct strategies arising from different trait combinations. Species from less seasonal habitats may try to maintain water status through a drought to avoid desiccation. These species may maintain photosynthesis and growth and hence adapted to have smaller but more numerous stomata, highly conductive larger xylem to transport adequate water, and deep roots to maintain access to water (Chitra‐Tarak et al., 2021; Reich, 2014; Reich et al., 2003). Species from more seasonal forests may have evolved to tolerate desiccation by decreasing photosynthesis to reduce water loss, with smaller stomata, and smaller xylem with lower conductivity (Reich et al., 2003). This strategy impedes growth but reduces the chance of embolism or hydraulic failure (Adams et al., 2017; Anderegg et al., 2015, 2016; Skelton et al., 2017).

Desiccation tolerant evergreen species from more seasonal forest should be resource‐conservative to prevent carbon starvation. Which should be reflected in features such as lower investment in below‐ground tissue (root mass fraction, RMF) to forage for water compared with desiccation avoiders (Lawson & Blatt, 2014; Rodríguez‐Ramírez et al., 2022), as well as other associated traits such as lower specific leaf area (SLA) and specific root length (length per unit mass, SRL), higher leaf dry matter content (LDMC) and stem tissue density (Poorter & Markesteijn, 2008). Alternatively, traits may not be coordinated and different trait combinations may offer equivalent responses to drought, with individual traits varying in their relationship to drought response (Laughlin et al., 2020). Easily measurable traits relevant to resource‐use may therefore help categorize species as avoiding or tolerating desiccation based on a whole‐plant evaluation of photosynthesis, growth and survival (Bartlett et al., 2012; Sun et al., 2020).

We assessed the relationship between traits and species' performance in dry vs. well‐watered conditions for seedlings of 16 tree species from a wet tropical forest region in a global biodiversity hotspot in southern India, the Western Ghats. Across the region, most seedlings establish during the monsoon, but then have to withstand dry periods varying from 3 to 9 months to persist. Given the range of dry months in this landscape, the seedlings must have a suite of traits evolved to tolerate or avoid desiccation. The species were chosen to represent a gradient in their adaptation to seasonal drought, climatic water deficit during the dry season (henceforth seasonality), based on a previous analysis of tree species distributions in the Western Ghats (Krishnadas et al., 2021). We asked:

1. Do species from less vs. more seasonal forests vary in their drought response?

We expected that species associated with greater seasonality would tolerate desiccation better than species from less seasonal forests, and therefore survive better but decrease photosynthesis and/or growth in drought vs. well‐watered conditions.

2 How well do individual traits explain interspecific variation in drought response?

We expected that species with higher SLA, lower LDMC, higher root mass fraction (RMF) or deeper roots, larger stomata and wider xylem, suite of traits found in plants adapted to wetter habitats, would maintain better function (photosynthesis and growth) early in drought, but survive less than species with lower SLA, higher LDMC, and lower RMF or shallow roots, smaller stomata, and narrower vessels.

3 Do distinct trait combinations characterize species that avoid or tolerate desiccation?

We expect that traits associated with conservative water‐use or safe hydraulic strategy, e.g., thicker leaves, smaller stomata and xylem, would correspond with responses characteristic of desiccation tolerant species adapted to more seasonal habitats—decreased photosynthesis and growth. Species adapted to less seasonal habitats, with thinner leaves and larger stomata or xylem would avoid desiccation by functioning at similar levels in drought compared with watered conditions. We also expect that species with higher SLA, lower LDMC, deeper roots and higher RMF would avoid desiccation. Species with lower SLA, higher LDMC, shallow roots and lower RMF were expected to tolerate desiccation.

## 2 METHODS

We conducted a dry‐down experiment in the greenhouse to gauge the response of seedlings to seasonal drought. Hence, our experiment represented a scenario where seedlings growing in a limited volume of soil were exposed to sudden drought and results should be interpreted accordingly. Seeds of 16 different tree species (Table A1) were obtained from two locations, Valparai in southern Western Ghats and Sirsi in northern Western Ghats, representing less and more seasonal climates respectively. Sirsi has higher mean annual rainfall but more negative climatic water deficit (CWD) (Sirsi: Mean annual precipitation (MAP) = 3425 mm/year, number of dry months: 6, CWD = −826.9 mm/year; Valparai: MAP = 2169.3 mm/year, number of dry months: 4, CWD = −396.51 mm/year, Source: Chave et al., 2014; Fick & Hijmans, 2017), which is a measure of both length and magnitude of drought stress on plants (Stephenson, 1998) and characterizes seasonal drought conditions well (Krishnadas et al., 2021).

### 2.1 Greenhouse experiment

Between July and November 2019, seeds were germinated in germination trays containing a 2:1 mixture of all‐purpose soil and cocopeat at the greenhouse at the National Centre of Biological Sciences (Bengaluru, India). Because species fruited at different times, had recalcitrant seeds, and germination times varied among species, we planted species for germination whenever seeds became available. Seeds were watered every alternate day and monitored for germination. Once germinants lost their cotyledon leaves and developed at least four mature leaves they were transplanted into PVC pipes of diameter 15 cm and depth of 45 cm (Pot volume = 7.95 L). The PVC columns were filled with a prepared soil mixture (all‐purpose soil: sand: cocopeat: compost = 1:1:1:0.1) and randomly assigned to six blocks within the greenhouse. Seedlings within a block were randomly assigned to drought and control treatments. Each treatment per block had an equal number of individuals of a species, but numbers differed among species depending on seedling availability. To maintain a consistent post‐germination age at which seedlings were subject to drought, species were included in the experiment as their seedlings reached four‐leaf stage. All new seedlings were allowed to acclimate in their pots for 2 weeks and watered every 2 days to avoid transplant shock. Seedlings that perished during this period were replaced and included after acclimation. In total, we had 434 seedlings in the experiment with 1–3 individuals per species per treatment per block.

After the acclimation period, we stopped watering the seedlings in the drought treatment for each block. Seedlings in the control treatment were given 75–100 mL of water twice weekly to ensure a consistent level of soil moisture. The greenhouse was maintained at approximately 65% relative humidity and 26°C temperature. Initially, we planned the drought treatment to last 16 weeks to simulate the post‐monsoon seasonal drought that occurs from January through April in the Western Ghats. However, monitoring and measurements were disrupted due to the COVID‐19 lockdown. For 12 of the 16 species, we implemented the experiment for 16 weeks. For the remaining four species, control plants were watered by the greenhouse staff but data collection was not possible due to restricted access to the facility. We harvested these plants in the 12th week of their drought experiment when we got permission to access the facility. These 12 weeks still give us a reasonable estimate of interspecific variation in response to seasonal drought conditions.

For growth, stem height, leaf length and width (for the four largest leaves) and survival were recorded weekly for the first 8 weeks and then once a month till the plants were harvested. We calculated the growth rate at the end of 12 weeks for all individuals as follows: (final height – initial height)/time in weeks. Seedling final height was strongly correlated with initial height (*r* = 0.7, *t* = 20.8, *p* < .001), hence we calculated relative growth rates, standardized by initial height as follows: (final height – initial height)/(time in weeks*initial height). Photosynthesis was measured monthly using a LiCOR 6400XT on seedlings of all species from control and drought treatments. Photosynthesis rates were measured with a 2 × 3 cm^2^ leaf chamber. The chamber conditions were maintained at PAR 1500 μmol m^−2^ s^−1^, 65% humidity, 400 μmol s^−1^ flow rate, fan speed at 10,000 rpm. Measurements were taken between 0700 and 1200 h.

### 2.2 Trait measurements

For leaf trait measurements (listed in Table A2), 1–3 leaves each were collected from selected seedlings and immediately stored in a ziplock bag between moist tissue paper to make sure the leaves were fully saturated with water (Cornelissen et al., 2003). Five seedlings from the control group were selected for each species for trait measurements.

To measure SLA (cm^2^ g^−1^) and LDMC (μm mg^−1^) (Cornelissen et al., 2003), the fresh leaf area was measured using the CI‐202 leaf scanner (CID bio‐science). After measuring fresh weight, leaves were placed in paper bags and air‐dried for a week, following which they were dried at 60°C for 3 days to get dry leaf mass. SLA was calculated as the ratio of leaf area to its dry biomass. LDMC was calculated as the ratio of dry leaf biomass and fresh leaf biomass.

**FIGURE A1:**
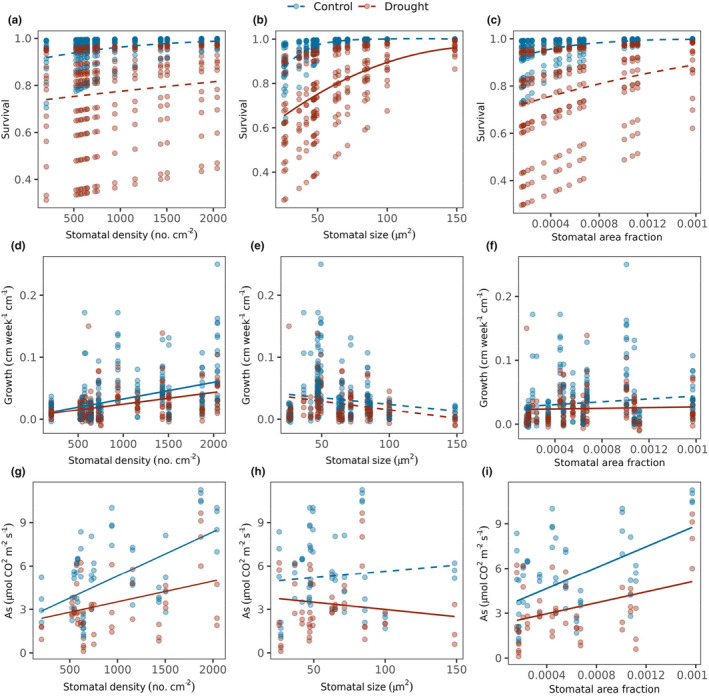
Comparing stomatal trait‐mediated seedling performance in drought. Seedling performance in drought vs. well‐watered conditions as a function of stomatal density, stomatal size and stomatal area fraction. Seedling survival under drought vs wel watered condition was modeled using Bernoulli errors as a function of (a) stomatal density, (b) stomatal size and (c) stomatal area fraction. Growth rate was modeled using Gaussian error structure for seedlings under drought vs. well‐watered conditions as a function of (d) stomatal density, (e) stomatal size and (f) stomatal area fraction. Photosynthesis rate was modeled using Gaussian error structure for seedlings under drought vs. well‐watered conditions as a function of (g) stomatal density, (h) stomatal size and (i) stomatal area fraction. For all the models, random intercepts for species and experimental block were used with and weightage of species. Lines indicate predicted response and points denote raw data. Continuous lines indicate a significant relationship at alpha <.05 and dashed lines indicate non‐significant results. Red lines and points denote drought conditions and blue denotes well‐watered conditions.

To measure stomatal traits, the leaf surface was wiped thoroughly to get rid of dust and excess moisture. Nail polish imprints of the underside were prepared and scanned under 40× magnification in a light microscope. For stomatal density (SD, number per cm^2^), four fields of vision were imaged from each imprint. The number of stomatal apertures was counted and the image area was measured. We chose three of the clearest stomates from each of the images and estimated stomatal size (SS, μm^2^) by drawing an ellipse around the stomata and stomatal length (SL, μm) was measured along the long axis of each stomata. We then calculated stomatal area fraction (SAF) for cm^2^ of leaf by taking a product of SD and SS (SD * SS * 10^−8^). Model results for SAF are similar to SD (Figure A1) and therefore we decided to use SD for further analysis and interpretation.

To measure vein density (VD, mm^−2^), whole leaves were immersed in petri dishes with cleaning bleach and monitored daily. Once the leaves lost all the chlorophyll, they were washed with distilled water and stained with methylene blue diluted 1:100 with distilled water. Whole leaf samples were fit on to clean slides and imaged under 20× magnification. Veins of all the orders were measured and density was calculated per mm^2^. Xylem diameter (XD, μm) was measured using an inch‐long section of the stem that was cut off and sectioned immediately into thin slices with a stainless‐steel blade. Five to ten of the thinnest sections were observed under the 40× light microscope and the diameter of 8–10 of the largest xylem vessels were measured using the line and circle tools of the software. All microscopic measurements were performed using a Primostar, Carl Zeiss microscope and images were taken using the camera accessory Axiocam‐105 mounted on the microscope. All images were analyzed to measure the stomatal, vein and xylem traits using the ZEISS ZEN2 (v1.0, RRID: SCR_013672).

To measure root traits, harvested roots were stored in a freezer until scanning. The root samples were taken out just before scanning and cleaned thoroughly to remove any traces of soil and dirt. The roots were scanned using an Epson Regent LA2400 scanner with a scale. The scans were analyzed using WinRHIZO (Regent Instruments Inc., Canada) for total root length (RL, mm.), diameter and root surface area (RA, mm^2^). SRL (cm g^−1^) was calculated as the ratio of the total root length and the root's dry biomass. RMF (g g^−1^) was calculated as the fraction of root dry mass and total plant dry biomass. Seasonality index (SI) of each species was derived from a previous analysis that estimated species' preference to more of less seasonal conditions based on their probability of occurrence across a landscape‐wide gradient of seasonal drought (Krishnadas et al., 2021). Positive value of seasonality index indicates that the species prefers less seasonal conditions, i.e., wet associated.

### 2.3 Statistical analysis

We used mixed‐effect models with appropriate error structures (see below) to test the impact of drought on survival, growth and photosynthesis. Preliminary models for growth showed that trends did not vary between growth rate ((final height – initial height) number of days between germination and harvest, Table A3) or relative growth rate (growth rate/initial height, Table A5) and we used the latter for interpretation since relative growth rates were standardized by initial height (See Table A4).

For the first question concerning differences in species response to drought in relation to their affinity for less vs. more seasonal areas, we modeled species' performance as a function of seasonality index, drought treatment, and their interaction. For question 2, we tested the role of each trait in governing drought response. We implemented separate models per trait, where species' performance was modeled as a function of their trait values, drought treatment, and drought‐trait interaction. In all models, we used species‐level trait means. Species ID and block ID were included as random intercepts. Survival was modeled using a Bernoulli error structure and Gaussian errors were assumed for growth and photosynthesis. Because species differed in the extent of variation in their response, we added a weighting term to separately model the variance per species. Trait models for SRL were implemented with and without the species *Actinodaphne malabarica*, which had small seedlings with very light roots and high SRL values that were an outlier compared with other species.

Model structure in Wilkinson‐Rogers notation:

$$\text{Performance variable}\sim \text{trait}+\text{treatment}+\text{trait}:\text{treatment}+\left(1|\text{species}\right)+\left(1|\text{block}\right),\text{weights}=\text{varIdent}\left(\text{form}=\sim 1|\text{species}\right)$$

**FIGURE A2:**
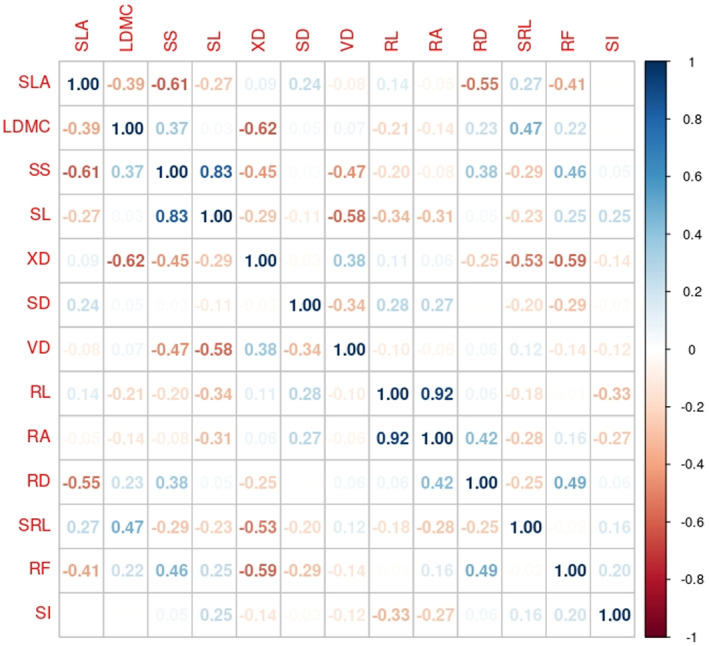
Plant trait correlation matrix: Matrix showing correlation coefficient (*r*) among plant traits. Color scale corresponding to the strength and sign of *r* is given on the right. Red denotes negative and blue denotes positive *r*. Intensity of the color corresponds to the strength of *r*. Abbreviations for the traits are given in Table A2.

Finally, to understand the impact of the overall trait‐based phenotype on performance during drought (question 3), we used a two‐step approach. First, we performed a pairwise correlation among traits (Figure A2) and a principal component analysis (PCA) on traits to obtain composite phenotypes defined by trait combinations. Then, we modeled individual survival, growth, and photosynthesis in control vs. drought plants using GLMM with species‐level intercepts and slopes. From these, we extracted each species' baseline performance (survival, growth, photosynthesis) in well‐watered conditions and the change in performance with drought and conducted a PCA on these six variables to get species' composite response to drought in relation to their baseline differences in growth, survival and photosynthesis. For both PCAs, we performed varimax rotation to simplify loadings for each factor. In the second step, to explore the correspondence between traits and performance, we performed Procrustean superimposition on the two PCA, using the axes that explained >75% of the variation in each PCA. Procrustes analysis checks the strength of association between two ordinations for a common object (species in our case), i.e., how well species positions in trait space matched their positions in performance space (Peres‐neto & Jackson, 2001; Rüger et al., 2018). Significance of the Procrustes correlation was assessed using permutation tests with 10,000 random iterations.

All analyses were performed using R version 3.4. We used packages nlme (Pinheiro et al., 2022) for mixed effects models with Gaussian error structure, lme4 (Bates et al., 2015) for models with Bernoulli error, FactoMineR (Lê et al., 2008) for PCA, and vegan (Oksanen et al., 2020) for Procrustes analysis.

## 3 RESULTS

### 3.1 Seasonality affiliation and drought response

**FIGURE 1:**
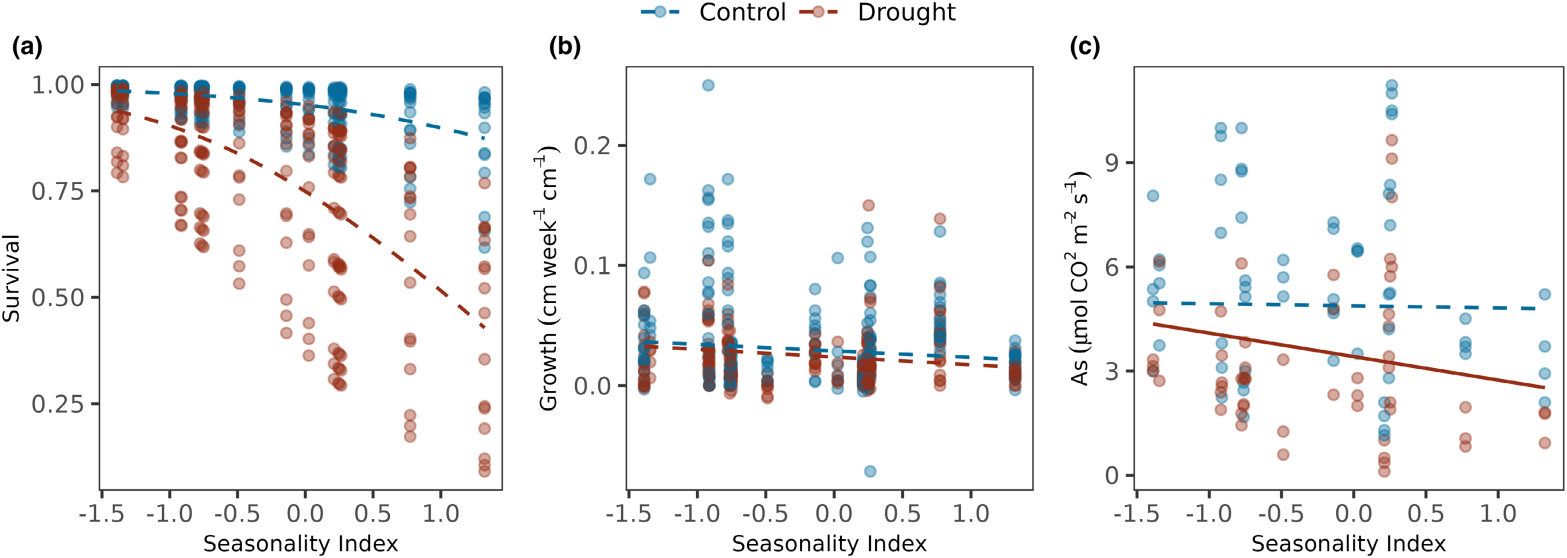
Seasonal affiliation‐mediated performance and survival in drought. Seedling survival and performance in drought vs well‐watered conditions are modeled as a function of their seasonal affiliation. Positive values of the *X*‐axis indicate affiliation to less seasonal conditions. (a) Results from generalized linear mixed effects model with binomial errors for seedling survival in drought vs well‐watered conditions as a function of their seasonal affiliations with random intercepts for species and experimental block. Results from linear mixed effects model for seedling relative growth rates (b) and photosynthesis rate (c) in drought vs well‐watered conditions as a function of their seasonal affiliation with random intercepts for species and experimental block and weightage of their seasonal index. Lines indicate predicted response and points denote raw data. Continuous lines indicate a significant relationship at alpha <.05 and dashed lines indicate non‐significant results. Red lines and points denote drought conditions and blue denotes wellwatered conditions.

Species' seasonality affiliation did not affect seedling survival (*β* = −.89, CI = −2.38 to 0.61, *z* = −1.16, *p* = .25, Figure 1a) but overall, seedlings had lower survival in drought vs well‐watered conditions (*β* = −2.17, CI = −2.95 to −1.39, *z* = −5.47, *p* < .05, Figure 1a). However, photosynthesis rates for species from less seasonal forests, i.e., larger values of seasonality index experienced larger decline under drought condition (*β* = −.45, CI = −0.82 to −0.08, *n* = 51, *t* = −2.46, *p* = .02, Figure 1c). Seasonality index did not explain interspecific variation in growth for plants in drought vs well‐watered conditions (*β* = −.001, CI = −0.003 to −0.002, *n* = 339, *t* = −0.64, *p* = .52, Figure 1b).

**FIGURE 2:**
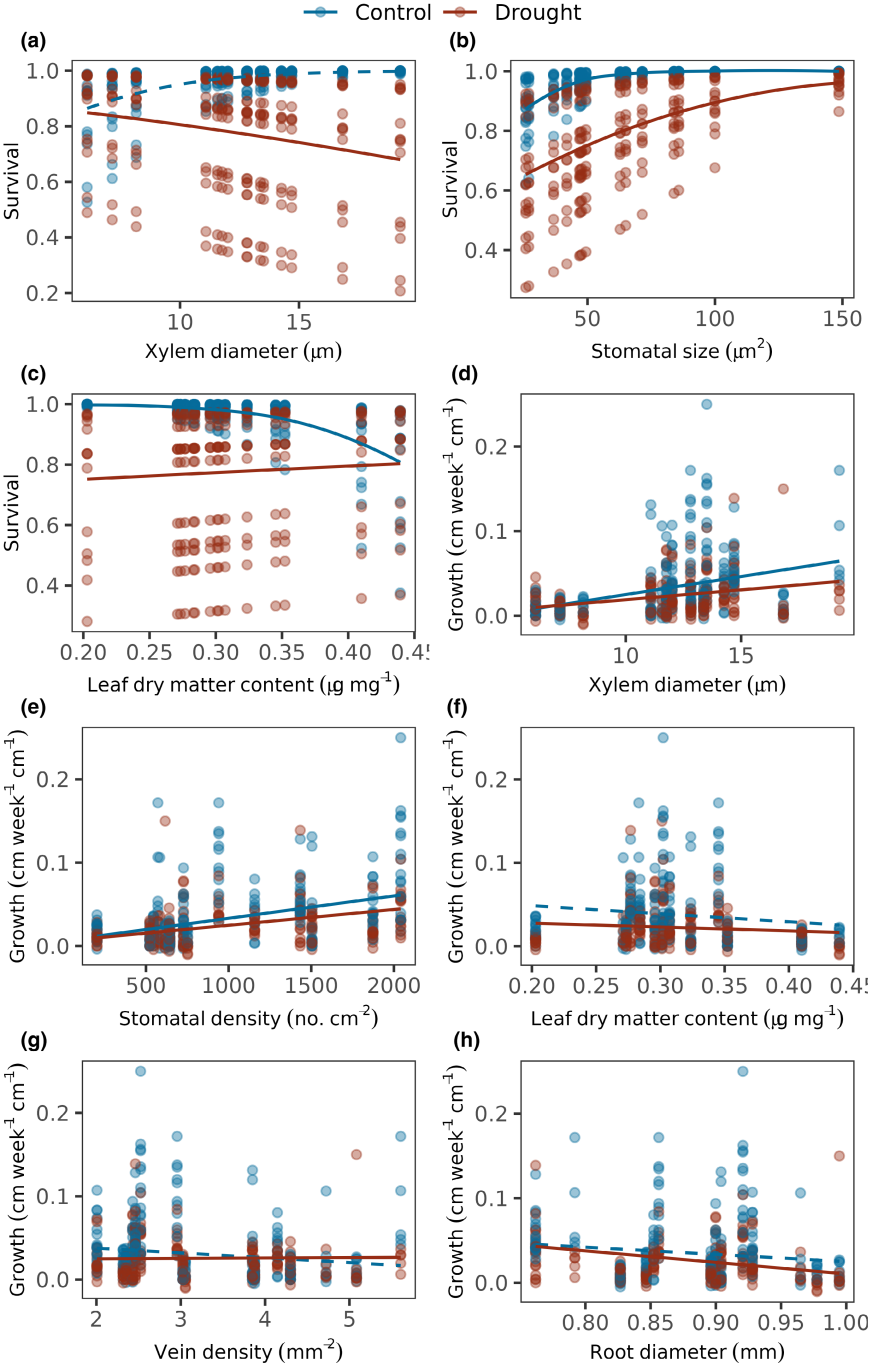
Trait‐mediated growth rate and survival in drought. Seedling survival in drought vs. well‐watered conditions as a function of (a) Xylem diameter, (b) Stomatal size and (c) Leaf dry matter content. Survival was modeled using Bernoulli errors with random intercepts for species and experimental blocks. Seedling growth under drought vs. well‐watered conditions as a function of (d) Xylem diameter, (e) Stomatal density, (f) Leaf dry matter content, (g) Vein density and (h) Root diameter. Growth rate was modeled using Gaussian errors with random intercepts for species and experimental block and weightage of species. Lines indicate predicted response and points denote raw data. Continuous lines indicate significant relationship at alpha <.05 and dashed lines indicate non‐significant results. Red lines and points denote drought and blue denotes well‐watered conditions.

Of the 12 traits we measured, traits related to leaf structural investment, anatomy of stomata and xylem best‐explained variation in drought response of seedlings. Species with larger xylem had lower survival in drought conditions when compared with well‐watered conditions (*β* = −1.3, CI = −2.14 to −0.45, *n* = 412, *z* = −3.0, *p* = .002, Figure 2a). Smaller stomata correlated with poorer survival in well‐watered conditions (*β* = 1.94, CI = 0.11 to 3.76, *n* = 412, *z* = 2.08, *p* = .04, Figure 2b), and survival also declined more steeply for species with smaller stomata in drought (*β* = −3.29, CI = −5.08 to −1.5, *n* = 412, *z* = −3.61, *p* < .001, Figure 2b). Although survival in well‐watered conditions decreased with higher LDMC, this difference disappeared under drought conditions (*β* = 1.2, CI = 0.41 to 2.0, *n* = 412, *z* = 2.97, *p* = .003, Figure 2c).

In well‐watered conditions, growth rates increased with increasing xylem diameter (*β* = .01, CI = 0.003 to 0.022, df = 13, *t* = 2.97, *p* = .01, Figure 2d) and higher stomatal density (*β* = .01, CI = 0.005 to 0.025, df = 14, *t* = 3.29, *p* < .05, Figure 2e). However, in drought condition, species with larger xylem and higher SD showed larger declines in growth but the effect sizes were weak (XD: *β* = −.01, CI = −0.008 to −0.004, df = 321, *t* = −5.09, *p* < .01; SD: *β* = −.004, CI = −0.009 to −0.0002, df = 339, *t* = −2.09, *p* = .04, Figure 2d,e). Species with lower LDMC and VD grew less under drought when compared with well‐watered condition. However with increasing values for both the traits, growth rates became more similar in well‐watered and drought conditions (LDMC: *β* = .003, CI = 0.001 to 0.004, df = 321, *t* = 3.37, *p* < .05, Figure 2f; VD: *β* = .01, CI = 0.003 to 0.009, df = 339, *t* = 4.31, *p* < .05, Figure 2g). Species with larger RD showed greater decline in growth under drought (*β* = −.003, CI = −0.006 to −0.0001, df = 296, *t* = −2.04, *p* = .04, Figure 2h).

**FIGURE 3:**
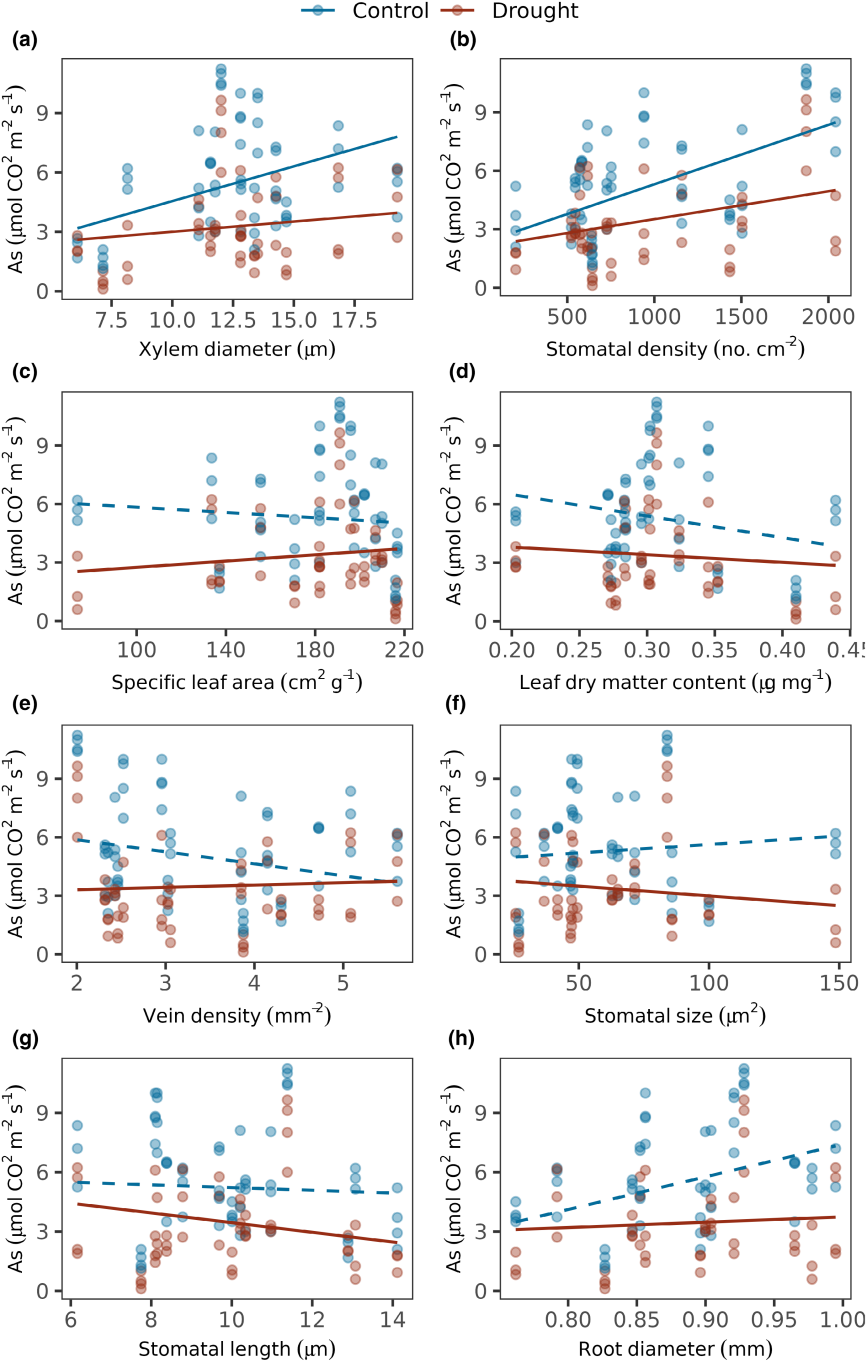
Trait‐mediated photosynthesis performance in drought. Seedling photosynthesis rates in drought vs. well‐watered conditions as a function of traits. Photosynthesis rate was modeled using Gaussian error structure for seedlings under drought vs. well‐watered conditions as a function of (a) Xylem diameter, (b) Stomatal density, (c) Specific leaf area, (d) Leaf dry matter content, (e) Vein density, (f) Stomatal Size, (g) Stomatal length and (h) Root diameter with random intercepts for species and experimental block and weightage of species. Lines indicate predicted response and points denote raw data. Continuous lines indicate a significant relationship at alpha < .05 and dashed lines indicate non‐significant results. Red lines and points denote drought conditions and blue denotes well‐watered conditions. As. denotes the Photosynthesis rate.

Physiological function (photosynthesis) increased with larger xylem (*β* = 1.14, CI = 0.08 to 2.19, df = 13, *t* = 2.3, *p* = .04, Figure 3a) and greater stomatal density (*β* = 1.6, CI = 0.65 to 2.49, df = 14, *t* = 3.65, *p* < .05, Figure 3b) as expected, and both slopes decreased in drought conditions (XD: *β* = −.8, CI = −1.01 to −0.60, df = 49, *t* = −7.73, *p* < .05; SD: *β* = −.83, CI = −1.22 to −0.45, df = 51, *t* = −4.37, *p* < .05, Figure 3a,b). SLA did not explain overall differences in photosynthetic rates of species in well‐watered conditions, but in drought, photosynthesis rates increased with SLA (*β* = .53, CI = 0.17 to 0.89, df = 49, *t* = 2.98, *p* < .05, Figure 3c), as well as with increasing LDMC (*β* = .4, CI = 0.15 to 0.64, df = 49, *t* = 3.2, *p* < .05, Figure 3d). Drought led to larger proportional decline in photosynthesis for species with lower vein density (*β* = .79, CI = 0.40 to 1.18, df = 51, *t* = 4.1, *p* < .05, Figure 3e). Additionally, photosynthesis decreased with larger and longer stomata in dry conditions (SS: *β* = −.55, CI = −0.86 to −0.24, df = 49, *t* = −3.6, *p* < .001, Figure 3f; SL: *β* = −.37, CI = −0.72 to −0.01, df = 49, *t* = −2.08, *p* = .04, Figure 3g).

Photosynthetic performance under drought was not explained by root area, length and SRL. Yet, under well‐watered conditions, photosynthesis increased with greater root area, longer roots and higher SRL (RA: *β* = 1.36, CI = 0.47 to 2.26, df = 12, *t* = 3.32, *p* < .05; RL: *β* = 1.12, CI = 0.13 to 2.11, df = 12, *t* = 2.48, *p* = .03; SRL: *β* = −.97, CI = −1.10 to 0.07, df = 11, *t* = −2.06, *p* = .06; Table A6h,i,k). Species with thicker roots had higher photosynthetic rates in well‐watered conditions, but experienced larger proportional decline in photosynthesis than species with thinner roots under drought stress (*β* = −.89, CI = −1.28 to −0.50, df = 47, *t* = −4.6, *p* < .05, Figure 3h).

### 3.2 Multi‐trait phenotype and drought response

**FIGURE A3:**
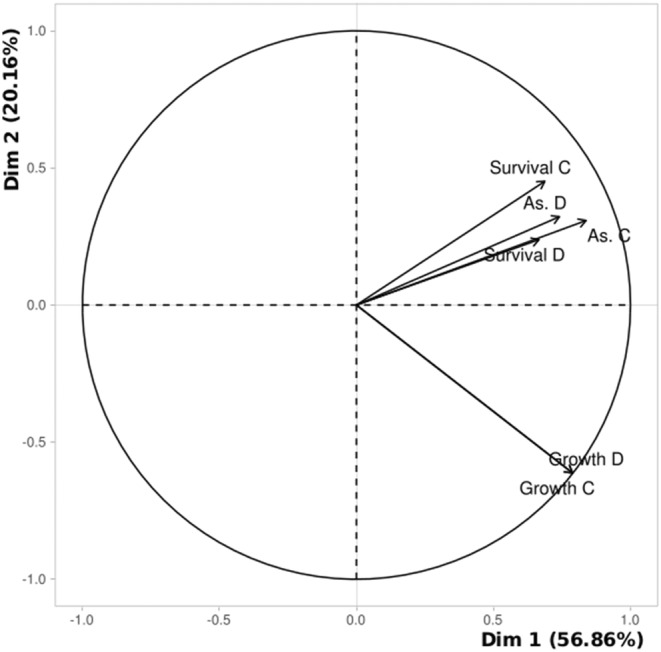
Seedling performance and survival in drought and well watered conditions. Principal Component Analysis decomposing variation among seedling performance and survival in drought and well watered condition. Direction of the arrows indicate the relative influence and the length of the arrow indicates the magnitude of influence of each predictor on seedling performance and survival. Survival indicates modeled survival probability, Growth indicates the modeled Relative Growth Rate and As. indicates modeled Photosynthetic rate. Suffix for each predictor indicates well watered (C) or drought (D) treatment.

Ordination for species performance under drought, the first two PCA axes explained 77.2% of the variation in performance among species‐level coefficients for survival, growth and photosynthesis (Figure A3 and Table A8). The first performance axis separated species according to their survival under drought vs. control conditions, with positive values indicating poor drought survival (Table A8). As a whole, the PCA showed that species that grew well also had higher photosynthetic rates and had higher survival in both drought and well‐watered conditions (Figure A3 and Table A8).

**FIGURE 4:**
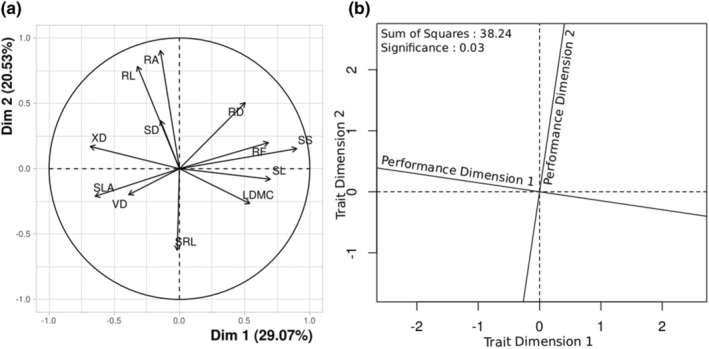
Concordance between trait and seedling drought performance. (a) PCA decomposing trait variation among seedlings. Traits included in PCA: Specific leaf area (SLA), Leaf dry matter content (LDMC), Stomatal density (SD), Xylem diameter (XD), Stomatal size (SS), Stomatal length (SL), Vein density (VD), Root mass fraction (RF), Specific root length (SRL), Root area (RA), Root diameter (RD), Total root length (RL). (b) Procrustes axes showing concordance (high axis overlap) between seedling trait (dashed lines) and drought performance (solid lines) ordinations. Inset: Sum of Squares of the loading displacement (lower value indicates higher overlap between axis); Significance from permutation test (<.05 means that similar results were obtained in more than 95% permutations).

After varimax rotation, PCA for traits required four dimensions to explain ~80% of the variation among species (Figure 4a, Table A10). Most axes had at least two traits with loadings >0.5. The first PCA axis separated species based on their stomatal, leaf structural and xylem traits and explained 29% variation. Positive values of axis 1 corresponded to lower SLA, larger stomates, higher leaf structural tissue, smaller xylem, and larger root mass fraction (Table S11). Axis 2 separated species based on water acquisition traits, explaining ~21% variation. Species with positive values on axis 2 had larger, longer and denser roots (Table A11). However, the third and fourth PCA axes reveal alternative combinations of measured traits. For instance, compared with axis 1, axis 3 comprised species with higher LDMC that also had higher SRL and smaller stomates. Axis 4 showed that, in contrast to axis 1, low SLA species could have smaller xylem and higher vein density (see Table A9).

Procrustes analysis between the ordinations of species performance (two axes) and traits (four axes) showed substantial concordance (sum of squares = 38.24, Figure 4b, *r* = .66, *p* = .03; permutation test for significance at alpha 0.05). This indicates that species' performance in drought conditions could be explained by composite phenotypes discerned from anatomical and morphological traits.

## 4 DISCUSSION

We found that drought reduced survival and growth for seedlings of 16 tree species from a wet tropical forest region in India (Western Ghats), but species varied substantially in their responses to drought. Interspecific variation in survival and growth with drought was primarily explained by anatomical traits that mediate water use and hydraulic safety. As expected, change in photosynthetic performance under drought stress correlated best with anatomical traits related to water use and gas exchange. Generally, species with fewer stomata and smaller xylem showed smaller declines in performance, growth and survival with drought. Growth constituted a distinct axis when compared with survival and photosynthesis for variation in species' drought response (Figure A3), but the overall variation in performance among species was explained by composite phenotypes determined by multiple traits. Species from more seasonal sites showed larger proportional decline in photosynthetic performance under drought (Figure 1c), but species affinity to seasonality conditions did not explain variation in drought response of growth or survival.

Different aspects of species' drought performance were regulated by different traits, but stomata and stem xylem traits correlated with all tested measures of drought performance, i.e., photosynthesis, growth and survival. Seedling survival and growth in well‐watered conditions improved with larger xylem and stomata (Figure 1a,b). In well‐watered conditions therefore, survival improves with traits that allow moving larger volumes of water for carbon assimilation. Accordingly, species with larger xylem also had higher photosynthetic rates (Figure 3a). The benefit of larger xylem for survival diminished in dry conditions (Figure 2a), consistent with larger xylem being more vulnerable to hydraulic failure due to embolism and cavitation, as known from mature trees (Hoffmann et al., 2011; Santiago et al., 2018; Skelton et al., 2015).

Tighter stomatal packing improved photosynthesis and growth when water was not limited (Medeiros et al., 2019). As with xylem size, this advantage reversed in drought conditions, when species with larger, more densely packed stomates experienced substantially larger declines in photosynthesis (Figure 3b,f,g) and growth (Figure 2e). Larger stomata imply higher conductance but also greater water loss, as with more numerous stomata (Lawson & Blatt, 2014), likely resulting in greater water loss, which becomes problematic during water limitation. Note that stomatal area fraction (SAF = SD*SS) showed results consistent with stomatal density (Figure A3), but smaller stomatal size did not correlate with higher stomatal density, suggesting that stomatal size and density form different axes to regulate water‐use (Anderegg et al., 2018).

Species that proportionally invested in longer roots, i.e., lower specific root length, had better survival when water was plenty, but experienced larger decline in survival in drought conditions (Table A7i). This is consistent with resource‐acquisitive being disadvantaged in stressful conditions, but this was not reflected in photosynthetic performance as expected. Photosynthetic rates did not vary with root mass fraction, or the length and area of roots in either well‐watered or drought conditions, even as average photosynthetic rates declined with water scarcity. We thus found no support for the expectation that investing in below ground tissue can help maintain growth during drought by improving access to water (Chitra‐Tarak et al., 2018; Joslin et al., 2000; Paz et al., 2015; Poorter & Markesteijn, 2008). It is possible that our findings may be an artifact of pot experiments where plants can only explore a finite soil volume, which might reduce the relevance of root traits in acquiring water.

In general, trait values that supported better growth in well‐watered conditions resulted in larger declines in growth with drought, suggesting a safety‐efficiency trade‐off with respect to water use (Choat et al., 2012). Species with larger xylem, suggesting higher hydraulic conductivity, also decreased photosynthesis during drought, but in this case, reduced photosynthesis translated to greater mortality suggesting carbon starvation with drought. Variability of trait effects on different aspects of performance was unsurprising when considering that survival and photosynthesis comprised distinct axes when compared with the growth for variation among species (see PCA of species coefficients for performance variables, Figure A10). Moreover, different combinations of traits were possible and required to explain interspecific variation in the overall drought response represented by changes in survival, growth, and photosynthesis.

A few caveats temper interpretation of our results. Extrapolating from pot experiments to performance in field conditions merits caution. Plant size affects soil water depletion and transpiration rates when growing in a limited volume of soil, influencing comparisons among species (Comita & Engelbrecht, 2014). Initial height, however, did not predict growth rates or drought responses in our study. Second, in closed canopy forests, shading affects air temperature and vapor pressure deficit (VPD), which can modulate the effects of water availability (Kupers et al., 2019). In addition, larger trees often transport water from deeper layers towards the surface (hydraulic redistribution) and increase water availability for seedlings to buffer drought stress. Alternatively, drought in natural settings may increase mortality due to other stressors such as nutrient limitation or herbivory, which we did not assess. Transplant experiments in the field will be needed to understand ‘real world’ implications of drought for seedling performance (Comita & Engelbrecht, 2014).

## 5 CONCLUSION

We found that anatomical traits related to xylem and stomata played a more prominent role than commonly used traits (e.g., SLA, LDMC, Greenwood et al., 2017) in explaining the drought response of tree seedlings from tropical humid forests of south Asia. These anatomical traits are directly involved in water movement, gas exchange and hydraulic safety (Brodribb et al., 2003; Brodribb & Holbrook, 2003; Sterling, 2005), and may serve as effective proxies to link hydraulic performance and whole‐plant response of tree seedlings to drought, allowing relatively rapid comparisons among species in field conditions and making generalizable predictions. Overall, drought conditions equalized performance differences seen in well‐watered conditions or advantaged some species based on their traits. Increased frequency of intra‐seasonal droughts or severity of the dry season due to global environmental change may thus influence which tree species regenerates successfully and accordingly alter functional composition of humid tropical forests (Chou et al., 2013).

## AUTHOR CONTRIBUTIONS

**Rishiddh Jhaveri:** Data curation (equal); formal analysis (equal); investigation (equal); validation (equal); visualization (equal); writing – original draft (equal); writing – review and editing (equal). **Lakshmipriya Cannanbilla:** Data curation (equal); formal analysis (equal); project administration (equal). **K. S. Arpitha Bhat:** Data curation (equal); project administration (equal). **Mahesh Sankaran:** Investigation (equal); resources (equal); validation (equal); writing – review and editing (equal). **Meghna Krishnadas:** Conceptualization (equal); data curation (equal); formal analysis (equal); funding acquisition (equal); investigation (equal); methodology (equal); project administration (equal); resources (equal); supervision (equal); validation (equal); visualization (equal); writing – original draft (equal); writing – review and editing (equal).

## FUNDING INFORMATION

NCBS campus fellowship (through TIFR and the Dept. of Atomic Energy, Government of India); National Geographic Society explorer level‐I grant.

## CONFLICT OF INTEREST STATEMENT

The authors declare no conflict of interest.

### OPEN RESEARCH BADGES

This article has earned an Open Data badge for making publicly available the digitally‐shareable data necessary to reproduce the reported results. The data is available at doi: 10.5061/dryad.4j0zpc8n1.

## Supporting information


Data S1.

## Data Availability

Data used for all the analysis in this study is submitted as supplementary material with the manuscript.

## Appendix

**TABLE A1:** Species of seedlings included in experiment.

S.no	Species	Species code	Seasonality index	Seed location
1	*Actinodaphne malabarica*	ACTMAL	0.21	Valparai
2	*Artocarpus heterophyllus*	ARTHET	0.26	Sirsi
3	*Aporosa lindleyana*	APOLIN	*−*0.91	Sirsi
4	*Diospyros angustifolia*	DIOANG	*−*0.76	Valparai
5	*Elaeocarpus serratus*	ELASER	*−*0.14	Valparai
6	*Elaeocarpus tuberculatus*	ELATUB	0.77	Valparai
7	*Garcinia indica*	GARIND	*−*0.75	Sirsi
8	*Garcinia talbotii*	GARTAL	*−*0.49	Sirsi
9	*Holigarna grahamii*	HOLGRA	*−*1.35	Sirsi
10	*Myristica malabarica*	MYRMAL	0.25	Valparai
11	*Olea dioica*	OLEDIO	*−*1.39	Sirsi
*12*	*Palaquium ellipticum*	PALELL	1.32	Valparai
13	*Persea macrantha*	PERMAC	0.03	Valparai
14	*Syzygium caryophyllatum*	SYZCAR	*−*0.92	Sirsi
15	*Syzygium cumini*	SYZCUM	−0.78	Sirsi
16	*Syzygium gardneri*	SYZGAR	0.24	Valparai

*Note*: Species code is the combination of first three letters of the Genus followed by first three letters of the Species. These codes are used to refer to each species in the main text. Higher seasonality index values indicate species associated to more seasonal forest (Krishnadas et al., 2021). Seeds used for the experiment were collected from two locations, Sirsi (Karnataka state, India) and Valparai (Tamil Nadu state, India).

**TABLE A2:** List of traits quantified in the study and their abbreviations as used in the manuscript. SI values were taken from Krishnadas et al. (2021).

Trait	Abbreviation
Specific leaf area	SLA
Leaf dry matter content	LDMC
Stomatal density	SD
Xylem diameter	XD
Stomatal size	SS
Stomatal length	SL
Stomatal area fraction	SAF
Vein density	VD
Root mass fraction	RMF
Specific root length	SRL
Root area	RA
Root diameter	RD
Total root length	RL
Seasonality Index	SI

**TABLE A3:** Trait‐mediated growth rate in drought.

(a) Growth Rate ~ SLA * Treatment (Drought/Control)					
Conditional *R* Squared = .1418	*β*	Std. Error	*t*‐Value	*p*‐Value	DF
Intercept	.46	0.08	5.73	.00	321
SLA	.11	0.07	1.52	.15	13
Treatment (Drought)	−.19	0.03	−6.35	.00	321
SLA: Treatment (Drought)	−.03	0.03	−1.09	.28	321

(b) Growth Rate ~ LDMC * Treatment (Drought/Control)					
Conditional *R* Squared = .0928	*β*	Std. Error	*t*‐Value	*p*‐Value	DF
Intercept	.45	0.08	5.34	.00	321
LDMC	−.06	0.08	−0.69	.50	13
Treatment (Drought)	−.19	0.03	−6.34	.00	321
LDMC: Treatment (Drought)	.03	0.03	0.85	.40	321

(c) Growth Rate ~ XD * Treatment (Drought/Control)					
Conditional *R* Squared = .3474	*β*	Std. Error	*t*‐Value	*p*‐Value	DF
Intercept	.36	0.06	5.84	.00	321
XD	.16	0.06	2.81	.02	13
Treatment (Drought)	−.09	0.01	−7.41	.00	321
XD: Treatment (Drought)	−.05	0.01	−6.03	.00	321

(d) Growth Rate ~ VD * Treatment (Drought/Control)					
Conditional *R* Squared = .0554	*β*	Std. Error	*t*‐Value	*p*‐Value	DF
Intercept	.32	0.07	4.91	.00	339
VD	−.06	0.06	−0.90	.38	14
Treatment (Drought)	−.05	0.01	−5.35	.00	339
VD: Treatment (Drought)	.06	0.01	5.39	.00	339

(e) Growth Rate ~ SD * Treatment (Drought/Control)					
Conditional *R* Squared = .2089	*β*	Std. Error	*t*‐Value	*p*‐Value	DF
Intercept	.44	0.08	5.86	.00	339
SD	.17	0.08	2.15	.05	14
Treatment (Drought)	−.18	0.03	−6.33	.00	339
SD: Treatment (Drought)	−.08	0.03	−2.99	.00	339

(f) Growth Rate ~ SL * Treatment (Drought/Control)					
Conditional *R* Squared = .1692	*β*	Std. Error	*t*‐Value	*p*‐Value	DF
Intercept	.43	0.08	5.47	.00	321
SL	−.14	0.08	−1.88	.08	13
Treatment (Drought)	−.19	0.03	−6.41	.00	321
SL: Treatment (Drought)	.08	0.03	2.65	.01	321

(g) Growth Rate ~ SS * Treatment (Drought/Control)					
Conditional *R* Squared = .1546	*β*	Std. Error	*t*‐Value	*p*‐Value	DF
Intercept	.44	0.08	5.63	.00	321
SS	−.13	0.07	−1.82	.09	13
Treatment (Drought)	−.19	0.03	−6.37	.00	321
SS: Treatment (Drought)	.06	0.03	1.92	.06	321

(h) Growth Rate ~ RA * Treatment (Drought/Control)					
Conditional *R* Squared = .0635	*β*	Std. Error	*t*‐Value	*p*‐Value	DF
Intercept	.38	0.07	5.15	.00	296
RA	.04	0.08	0.59	.57	12
Treatment (Drought)	−.09	0.01	−7.15	.00	296
RA: Treatment (Drought)	−.01	0.01	−0.87	.38	296

(i) Growth Rate ~ RL * Treatment (Drought/Control)					
Conditional *R* Squared = .1365	*β*	Std. Error	*t*‐Value	*p*‐Value	DF
Intercept	.38	0.07	5.36	.00	296
RL	.08	0.07	1.11	.29	12
Treatment (Drought)	−.08	0.01	−7.17	.00	296
RL: Treatment (Drought)	.00	0.01	−0.25	.80	296

(j) Growth Rate ~ RD * Treatment (Drought/Control)					
Conditional *R* Squared = .1790	*β*	Std. Error	*t*‐Value	*p*‐Value	DF
Intercept	.09	0.17	0.54	.59	296
RD	−.20	0.16	−1.28	.23	12
Treatment (Drought)	−.20	0.03	−7.20	.00	296
RD: Treatment (Drought)	−.05	0.03	−1.67	.10	296

(k) Growth Rate ~ SRL * Treatment (Drought/Control)					
Conditional *R* Squared = .1198	*β*	Std. Error	*t*‐Value	*p*‐Value	DF
Intercept	.41	0.08	5.33	.00	266
SRL	−.07	0.08	−0.86	.41	11
Treatment (Drought)	−.11	0.01	−7.75	.00	266
SRL: Treatment (Drought)	.00	0.02	0.03	.98	266

(l) Growth Rate ~ RF * Treatment (Drought/Control)					
Conditional *R* Squared = .3712	*β*	Std. Error	*t*‐Value	*p*‐Value	DF
Intercept	.45	0.06	7.92	.00	321
RF	−.29	0.05	−5.75	.00	13
Treatment (Drought)	−.19	0.03	−6.73	.00	321
RF: Treatment (Drought)	.18	0.03	6.33	.00	321

*Note*: List of model coefficients for seedling growth rate under drought vs. well‐watered conditions as a function of traits. Sub‐tables *a–l* refer to different models with each of 12 traits (refer to Table A2 for trait abbreviation). Growth rate was modeled using Gaussian errors with random intercepts for species and experimental block and weightage of species. First row of each sub‐table lists model response and explanatory variables with appropriate relationship. Model Conditional *R*‐squares are given in the second row of each sub‐table. Model co‐efficients (*β*), standard error (Std. Error), *t*‐statistics (*t*‐value), *p*‐value and degree of freedom (DF) are listed in columns in rows corresponding to each explanatory variable.

**TABLE A4:** Seasonal affiliation‐mediated performance and survival in drought.

(a) Survival (1/0) ~ Seasonality Index * Treatment (Drought/Control)				
Conditional *R* Squared = .3355	*β*	Std. Error	*z* Value	*p* Value
Intercept	3.65	0.59	6.15	.00
Seasonality Index	−.89	0.76	−1.16	.25
Treatment (Drought)	−2.17	0.40	−5.47	.00
Seasonality Index: Treatment (Drought)	−.46	0.60	−0.77	.44

(b) Relative Growth Rate ~ Seasonality Index * Treatment (Drought/Control)					
Conditional *R* Squared = .05	*β*	Std. Error	*t*‐Value	*p*‐Value	DF
Intercept	.030	0.01	5.73	.00	339
Seasonality Index	−.004	0.01	−0.77	.45	14
Treatment (Drought)	−.005	0.00	−3.89	.00	339
Seasonality Index: Treatment (Drought)	−.001	0.00	−0.64	.52	339

(c) Photosynthesis Rate ~ Seasonality Index * Treatment (Drought/Control)					
Conditional *R* Squared = .25	*β*	Std. Error	*t*‐Value	*p*‐Value	DF
Intercept	4.896	0.52	9.41	.00	51
Seasonality Index	−.045	0.51	−0.09	.93	14
Treatment (Drought)	−1.305	0.18	−7.24	.00	51
Seasonality Index: Treatment (Drought)	−.452	0.18	−2.46	.02	51

*Note*: Seedling survival and performance in drought vs well‐watered conditions are modeled as a function of their seasonal affiliation. (a) Results from generalized linear mixed effects model with binomial error for seedling survival in drought vs well‐watered conditions as a function of their seasonal affiliations with random intercepts for species and experimental block. Results from linear mixed effects model for seedling relative growth rates (b) and photosynthesis rate (c) in drought vs well‐watered conditions as a function of their seasonal affiliation with random intercepts for species and experimental block and weightage of their seasonal index. First row of each sub‐table lists model response and explanatory variables with appropriate relationship. Model Conditional *R*‐squares are given in the second row of each sub‐table. Model co‐efficients (*β*), standard error (Std. Error), *t*‐statistics (*t*‐value), *z*‐statistics (*z* Value), *p*‐value and degree of freedom (DF) are listed in columns in rows corresponding to each explanatory variable.

**TABLE A5:** Trait‐mediated relative growth rate in drought.

(a) Relative Growth Rate ~ SLA * Treatment (Drought/Control)					
Conditional *R* squared = .10	*β*	Std. Error	*t*‐Value	*p*‐Value	DF
Intercept	.03	0.01	6.44	.00	321
SLA	.01	0.00	1.33	.21	13
Treatment (Drought)	−.01	0.00	−5.21	.00	321
SLA: Treatment (Drought)	.00	0.00	1.81	.07	321

(b) Relative Growth Rate ~ LDMC * Treatment (Drought/Control)					
Conditional *R* Squared = .06	*β*	Std. Error	*t*‐Value	*p*‐Value	DF
Intercept	.03	0.01	6.03	.00	321
LDMC	−.01	0.01	−1.03	.32	13
Treatment (Drought)	−.01	0.00	−5.83	.00	321
LDMC: Treatment (Drought)	.00	0.00	3.37	.00	321

(c) Relative Growth Rate ~ XD * Treatment (Drought/Control)					
Conditional *R* Squared = .21	*β*	Std. Error	*t*‐Value	*p*‐Value	DF
Intercept	.03	0.00	7.16	.00	321
XD	.01	0.00	2.97	.01	13
Treatment (Drought)	−.01	0.00	−6.90	.00	321
XD: Treatment (Drought)	−.01	0.00	−5.09	.00	321

(d) Relative Growth Rate ~ VD * Treatment (Drought/Control)					
Conditional *R* Squared = .05	*β*	Std. Error	*t*‐Value	*p*‐Value	DF
Intercept	.03	0.01	5.87	.00	339
VD	−.01	0.00	−1.13	.28	14
Treatment (Drought)	−.01	0.00	−4.82	.00	339
VD: Treatment (Drought)	.01	0.00	4.31	.00	339

(e) Relative Growth Rate ~ SD * Treatment (Drought/Control)					
Conditional *R* Squared = .28	*β*	Std. Error	*t*‐Value	*p*‐Value	DF
Intercept	.03	0.00	7.90	.00	339
SD	.01	0.00	3.29	.01	14
Treatment (Drought)	−.01	0.00	−4.46	.00	339
SD: Treatment (Drought)	.00	0.00	−2.09	.04	339

(f) Relative Growth Rate ~ SL * Treatment (Drought/Control)					
Conditional *R* Squared = .12	*β*	Std. Error	*t*‐Value	*p*‐Value	DF
Intercept	.03	0.01	6.23	.00	321
SL	−.01	0.00	−1.46	.17	13
Treatment (Drought)	−.01	0.00	−5.10	.00	321
SL: Treatment (Drought)	.00	0.00	−1.31	.19	321

(g) Relative Growth Rate ~ SS * Treatment (Drought/Control)					
Conditional *R* Squared = .10	*β*	Std. Error	*t*‐Value	*p*‐Value	DF
Intercept	.03	0.01	6.34	.00	321
SS	−.01	0.00	−1.36	.20	13
Treatment (Drought)	−.01	0.00	−5.36	.00	321
SS: Treatment (Drought)	.00	0.00	−1.95	.05	321

(h) Relative Growth Rate ~ SAF * Treatment (Drought/Control)					
Conditional *R* Squared = .06	*β*	Std. Error	*t*‐Value	*p*‐Value	DF
Intercept	.03	0.01	6.08	.00	321
SAF	.00	0.01	0.84	.42	13
Treatment (Drought)	−.01	0.00	−5.81	.00	321
SAF: Treatment (Drought)	.00	0.00	−2.36	.02	321

(I) Relative Growth Rate ~ RA * Treatment (Drought/Control)					
Conditional *R* Squared = .05	*β*	Std. Error	*t*‐Value	*p*‐Value	DF
Intercept	.04	0.01	5.99	.00	296
RA	.00	0.01	0.62	.55	12
Treatment (Drought)	−.01	0.00	−6.59	.00	296
RA: Treatment (Drought)	.00	0.00	−2.19	.03	296

(j) Relative Growth Rate ~ RL * Treatment (Drought/Control)					
Conditional *R* Squared = .07	*β*	Std. Error	*t*‐Value	*p*‐Value	DF
Intercept	.04	0.01	6.08	.00	296
RL	.01	0.01	0.95	.36	12
Treatment (Drought)	−.01	0.00	−6.48	.00	296
RL: Treatment (Drought)	.00	0.00	−1.51	.13	296

(k) Relative Growth Rate ~ RD * Treatment (Drought/Control)					
Conditional *R* Squared = .11	*β*	Std. Error	*t*‐Value	*p*‐Value	DF
Intercept	.04	0.01	6.31	.00	296
RD	−.01	0.01	−0.99	.34	12
Treatment (Drought)	−.01	0.00	−6.44	.00	296
RD: Treatment (Drought)	.00	0.00	−2.04	.04	296

(l) Relative Growth Rate ~ SRL * Treatment (Drought/Control)					
Conditional *R* Squared = .09	*β*	Std. Error	*t*‐Value	*p*‐Value	DF
Intercept	.04	0.01	6.41	.00	266
SRL	−.01	0.01	−0.80	.44	11
Treatment (Drought)	−.01	0.00	−7.55	.00	266
SRL: Treatment (Drought)	.00	0.00	1.87	.06	266

(m) Relative Growth Rate ~ RF * Treatment (Drought/Control)					
Conditional *R* Squared = .26	*β*	Std. Error	*t*‐Value	*p*‐Value	DF
Intercept	.03	0.00	7.96	.00	321
RF	−.01	0.00	−3.01	.01	13
Treatment (Drought)	−.01	0.00	−4.68	.00	321
RF: Treatment (Drought)	.00	0.00	−1.33	.18	321

*Note*: List of model coefficients for seedling relative growth rate under drought vs. well‐watered conditions as a function of traits. Sub‐tables *a–l* refer to different models with each of twelve traits (refer to Table A2 for trait abbreviation). Relative growth rate was modeled using Gaussian errors with random intercepts for species and experimental block and weightage of species. First row of each sub‐table lists model response and explanatory variables with appropriate relationship. Model Conditional *R*‐squares are given in the second row of each sub‐table. Model co‐efficients (*β*), standard error (Std. Error), *t*‐statistics (*t*‐value), *p*‐value and degree of freedom (DF) are listed in columns in rows corresponding to each explanatory variable.

**TABLE A6:** Trait‐mediated Photosynthesis rate in drought.

(a) Phtotsynthesis Rate ~ SLA * Treatment (Drought/Control)					
Conditional *R* squared = .37	*β*	Std. Error	*t*‐Value	*p*‐Value	DF
Intercept	5.30	0.55	9.67	.00	49
SLA	−.24	0.52	−0.47	.65	13
Treatment (Drought)	−1.90	0.19	−10.23	.00	49
SLA: Treatment (Drought)	.53	0.18	2.98	.00	49

(b) Phtotsynthesis Rate ~ LDMC * Treatment (Drought/Control)					
Conditional *R* Squared = .42	*β*	Std. Error	*t*‐value	*p*‐value	DF
Intercept	5.29	0.54	9.81	.00	49
LDMC	−.61	0.53	−1.16	.27	13
Treatment (Drought)	−1.91	0.19	−10.33	.00	49
LDMC: Treatment (Drought)	.39	0.12	3.23	.00	49

(c) Phtotsynthesis Rate ~ XD * Treatment (Drought/Control)					
Conditional *R* Squared = .57	*β*	Std. Error	*t*‐Value	*p*‐Value	DF
Intercept	5.41	0.51	10.64	.00	49
XD	1.14	0.49	2.33	.04	13
Treatment (Drought)	−2.15	0.19	−11.50	.00	49
XD: Treatment (Drought)	−.80	0.10	−7.73	.00	49

(d) Phtotsynthesis Rate ~ VD * Treatment (Drought/Control)					
Conditional *R* Squared = .34	*β*	Std. Error	*t*‐Value	*p*‐Value	DF
Intercept	5.00	0.52	9.69	.00	51
VD	−.66	0.52	−1.27	.23	14
Treatment (Drought)	−1.52	0.14	−10.97	.00	51
VD: Treatment (Drought)	.79	0.19	4.09	.00	51

(e) Phtotsynthesis Rate ~ SD * Treatment (Drought/Control)					
Conditional *R* Squared = .61	*β*	Std. Error	*t*‐Value	*p*‐Value	DF
Intercept	5.13	0.42	12.09	.00	51
SD	1.57	0.43	3.65	.00	14
Treatment (Drought)	−1.69	0.15	−11.30	.00	51
SD: Treatment (Drought)	−.83	0.19	−4.37	.00	51

(f) Phtotsynthesis Rate ~ SL * Treatment (Drought/Control)					
Conditional *R* Squared = .35	*β*	Std. Error	*t*‐Value	*p*‐Value	DF
Intercept	5.23	0.55	9.58	.00	49
SL	−.14	0.53	−0.27	.79	13
Treatment (Drought)	−1.76	0.18	−9.91	.00	49
SL: Treatment (Drought)	−.37	0.18	−2.08	.04	49

(g) Phtotsynthesis Rate ~ SS * Treatment (Drought/Control)					
Conditional *R* Squared = .38	*β*	Std. Error	*t*‐Value	*p*‐Value	DF
Intercept	5.29	0.55	9.64	.00	49
SS	.26	0.52	0.50	.63	13
Treatment (Drought)	−1.91	0.17	−10.96	.00	49
SS: Treatment (Drought)	−.55	0.15	−3.57	.00	49

(h) Phtotsynthesis Rate ~ SAF * Treatment (Drought/Control)					
Conditional *R* Squared = .61	*β*	Std. Error	*t*‐Value	*p*‐Value	DF
Intercept	5.32	0.45	11.76	.00	49
SAF	1.47	0.46	3.20	.01	13
Treatment (Drought)	−2.01	.19	−10.79	.00	49
SAF: Treatment (Drought)	−.70	0.19	−3.58	.00	49

(i) Phtotsynthesis Rate ~ RA * Treatment (Drought/Control)					
Conditional *R* Squared = .66	*β*	Std. Error	*t*‐Value	*p*‐Value	DF
Intercept	5.64	0.42	13.42	.00	47
RA	1.36	0.41	3.32	.01	12
Treatment (Drought)	−2.20	0.23	−9.51	.00	47
RA: Treatment (Drought)	−.02	0.20	−0.11	.92	47

(j) Phtotsynthesis Rate ~ RL * Treatment (Drought/Control)					
Conditional *R* Squared = .62	*β*	Std. Error	*t*‐value	*p*‐Value	DF
Intercept	5.63	0.46	12.22	.00	47
RL	1.12	0.45	2.48	.03	12
Treatment (Drought)	−2.17	0.22	−9.75	.00	47
RL: Treatment (Drought)	.15	0.20	0.79	.44	47

(k) Phtotsynthesis Rate ~ RD * Treatment (Drought/Control)					
Conditional *R* Squared = .53	*β*	Std. Error	*t*‐Value	*p*‐Value	DF
Intercept	5.52	0.53	10.42	.00	47
RD	1.07	0.52	2.06	.06	12
Treatment (Drought)	−2.09	0.18	−11.63	.00	47
RD: Treatment (Drought)	−.89	0.19	−4.62	.00	47

(l) Phtotsynthesis Rate ~ SRL * Treatment (Drought/Control)					
Conditional *R* Squared = .59	*β*	Std. Error	*t*‐Value	*p*‐Value	DF
Intercept	5.99	0.47	12.72	.00	43
SRL	−0.97	0.47	−2.06	.06	11
Treatment (Drought)	−2.51	0.23	−11.09	.00	43
SRL: Treatment (Drought)	.23	0.21	1.08	.29	43

(m) Phtotsynthesis Rate ~ RF * Treatment (Drought/Control)					
Conditional *R* Squared = .40	*β*	Std. Error	*t*‐Value	*p*‐Value	DF
Intercept	5.27	0.54	9.80	.00	49
RF	−.28	0.54	−0.53	.61	13
Treatment (Drought)	−1.84	0.20	−9.16	.00	49
RF: Treatment (Drought)	−.33	0.27	−1.25	.22	49

*Note*: List of model coefficients for seedling photosynthesis rate under drought vs. well‐watered conditions as a function of traits. Sub‐tables *a–l* refer to different models with each of 12 traits (refer to Table A2 for trait abbreviation). Photosynthesis rate was modeled using Gaussian errors with random intercepts for species and experimental block and weightage of species. First row of each sub‐table lists model response and explanatory variables with appropriate relationship. Model Conditional *R*‐squares are given in the second row of each sub‐table. Model co‐efficients (*β*), standard error (Std. Error), *t*‐statistics (*t*‐value), *p*‐value and degree of freedom (DF) are listed in columns in rows corresponding to each explanatory variable.

**TABLE A7:** Trait‐mediated Seedling Survival in drought.

(a) Survival (1/0) ~ SLA * Treatment (Drought/Control)				
Conditional *R* squared = .40	*β*	Std. Error	*z* value	*p* value
Intercept	4.42	0.77	5.73	.00
SLA	−0.95	0.69	−1.38	.17
Treatment (Drought)	−2.57	0.51	−5.06	.00
SLA: Treatment (Drought)	0.80	0.56	1.44	.15

(b) Survival (1/0) ~ LDMC * Treatment (Drought/Control)				
Conditional *R* Squared = .43	*β*	Std. Error	*z* value	*p* Value
Intercept	4.81	0.83	5.82	.00
LDMC	−1.11	0.60	−1.84	.07
Treatment (Drought)	−2.91	0.57	−5.09	.00
LDMC: Treatment (Drought)	1.20	0.41	2.97	.00

(c) Survival (1/0) ~ SD * Treatment (Drought/Control)				
Conditional *R* Squared = .35	*β*	Std. Error	*z* Value	*p* Value
Intercept	4.09	0.67	6.15	.00
SD	.64	0.68	0.93	.35
Treatment (Drought)	−2.32	0.43	−5.39	.00
SD: Treatment (Drought)	−.46	0.53	−0.87	.38

(d) Survival (1/0) ~ XD * Treatment (Drought/Control)				
Conditional *R* Squared = .42	*β*	Std. Error	*z* Value	*p* Value
Intercept	4.62	0.80	5.79	.00
XD	1.00	0.61	1.63	.10
Treatment (Drought)	−2.69	0.54	−5.00	.00
XD: Treatment (Drought)	−1.30	0.43	−3.00	.00

(e) Survival (1/0) ~ VD * Treatment (Drought/Control)				
Conditional *R* Squared = .32	*β*	Std. Error	*z* Value	*p* Value
Intercept	4.05	0.60	6.78	.00
VD	−0.73	0.50	−1.46	.14
Treatment (Drought)	−2.33	0.45	−5.23	.00
VD: Treatment (Drought)	.45	0.40	1.11	.27

(f) Survival (1/0) ~ SL * Treatment (Drought/Control)				
Conditional *R* Squared = .40	*β*	Std. Error	*z* Value	*p* Value
Intercept	4.62	0.81	5.71	.00
SL	1.16	0.67	1.73	.08
Treatment (Drought)	−2.75	0.61	−4.54	.00
SL: Treatment (Drought)	−.79	0.56	−1.40	.16

(g) Survival (1/0) ~ SS * Treatment (Drought/Control)				
Conditional *R* Squared = .46	*β*	Std. Error	*z* Value	*p* Value
Intercept	5.06	1.02	4.95	.00
SS	1.94	0.93	2.08	.04
Treatment (Drought)	−3.29	0.91	−3.61	.00
SS: Treatment (Drought)	−1.31	0.87	−1.51	.13

(h) Survival (1/0) ~ SAF * Treatment (Drought/Control)				
Conditional *R* Squared = .39	*β*	Std. Error	*z* Value	*p* Value
Intercept	4.47	0.76	5.89	.00
SAF	1.08	0.76	1.43	.15
Treatment (Drought)	−2.61	0.57	−4.59	.00
SAF: Treatment (Drought)	−.67	0.64	−1.04	.30

(i) Survival (1/0) ~ RA * Treatment (Drought/Control)				
Conditional *R* Squared = .33	*β*	Std. Error	*z* Value	*p* Value
Intercept	3.93	0.68	5.82	.00
RA	.66	0.58	1.15	.25
Treatment (Drought)	−2.29	0.47	−4.83	.00
RA: Treatment (Drought)	−.03	0.44	−0.07	.95

(j) Survival (1/0) ~ RL * Treatment (Drought/Control)				
Conditional *R* Squared = .33	*β*	Std. Error	*z* Value	*p* Value
Intercept	3.85	0.66	5.87	.00
RL	.46	0.62	0.74	.46
Treatment (Drought)	−2.14	0.44	−4.81	.00
RL: Treatment (Drought)	.38	0.50	0.76	.45

(k) Survival (1/0) ~ RD * Treatment (Drought/Control)				
Conditional *R* Squared = .35	*β*	Std. Error	*z* Value	*p* Value
Intercept	4.07	0.71	5.71	.00
RD	.36	0.54	0.66	.51
Treatment (Drought)	−2.43	0.48	−5.10	.00
RD: Treatment (Drought)	−.36	0.38	−0.93	.35

(l) Survival (1/0) ~ SRL * Treatment (Drought/Control)				
Conditional *R* Squared = .46	*β*	Std. Error	*z* Value	*p* Value
Intercept	4.61	0.79	5.86	.00
SRL	−1.33	0.50	−2.64	.01
Treatment (Drought)	−3.06	0.61	−5.02	.00
SRL: Treatment (Drought)	.84	0.28	2.95	.00

(m) Survival (1/0) ~ RF * Treatment (Drought/Control)				
Conditional *R* Squared = .34	*β*	Std. Error	*z* Value	*p* Value
Intercept	4.00	0.65	6.14	.00
RF	−.27	0.67	−0.40	.69
Treatment (Drought)	−2.22	0.40	−5.58	.00
RF: Treatment (Drought)	.03	0.54	0.06	.95

*Note*: List of model coefficients for seedling survival probability under drought vs. well‐watered conditions as a function of traits. Sub‐tables *a–l* refer to different models with each of 12 traits (refer to Table A2 for trait abbreviation). Seedling survival probability was modeled using Bernoulli errors with random intercepts for species and experimental blocks. First row of each sub‐table lists model response and explanatory variables with appropriate relationship. Model Conditional *R*‐squares are given in the second row of each sub‐table. Model co‐efficients (*β*), standard error (Std. Error), *z*‐statistics (*z*. value) and *p*‐value are listed in columns in rows corresponding to each explanatory variable.

**TABLE A8:** PCA loadings for species performance model and survival model coefficients.

Model predictor	Dim. 1	Dim. 2
Predicted Growth C	0.79	−0.61
Predicted Growth D	0.79	−0.61
Predicted Photosynthesis C	0.84	0.31
Predicted Photosynthesis D	0.74	0.32
Predicted Survival C	0.69	0.45
Predicted Survival D	0.67	0.24

*Note*: Two Principal Component Analysis dimensions with three performance parameters in drought and well watered condition, explained ~77% variation among species. Suffix for each predictor indicates well watered (C) or drought (D) treatment.

**TABLE A9:** Species vice PCA loadings for performance model and survival model coefficients.

Species	Dim. 1	Dim. 2
*Syzygium cumini*	3.74	0.91
*Syzygium caryophyllatum*	3.09	−0.12
*Holigarna grahamii*	2.39	1.57
*Artocarpus heterophyllus*	1.97	0.13
*Elaeocarpus tuberculatus*	0.11	0.30
*Myristica malabarica*	−0.05	−0.30
*Garcinia talbotii*	−0.13	−1.26
*Olea dioica*	−0.17	−0.86
*Garcinia indica*	−0.18	−1.68
*Persea macrantha*	−0.41	−0.37
*Palaquium ellipticum*	−0.71	−0.77
*Syzygium gardneri*	−1.08	0.73
*Diospyros angustifolia*	−1.12	−1.84
*Elaeocarpus serratus*	−1.27	1.40
*Aporosa lindleyana*	−2.39	0.24
*Actinodaphne malabarica*	−3.79	1.93

*Note*: Two Principal Component Analysis dimensions with 16 species, explained ~77% variation among species performance in drought vs. well watered conditions.

**TABLE A10:** PCA loadings for species traits.

Traits	Dim. 1	Dim. 2	Dim. 3	Dim. 4
SLA	−0.65	−0.21	−0.13	0.53
LDMC	0.54	−0.27	0.54	0.20
SS	0.90	0.15	−0.31	0.00
SL	0.70	−0.08	−0.63	0.05
XD	−0.68	0.17	−0.37	−0.55
SD	−0.14	0.37	−0.18	0.58
VD	−0.39	−0.20	0.52	−0.62
RL	−0.32	0.78	0.22	0.30
RA	−0.15	0.90	0.32	0.14
RD	0.50	0.51	0.36	−0.30
SRL	−0.02	−0.62	0.57	0.42
RF	0.68	0.20	0.31	−0.09

*Note*: Four Principal Component Analysis dimensions with 12 traits explaining ~80% variation among species traits.

**TABLE A11:** Species vice PCA loadings for traits.

Species	Dim. 1	Dim. 2	Dim. 3	Dim. 4
*Garcinia talbotii*	5.59	−0.30	0.41	−1.01
*Palaquium ellipticum*	1.61	−1.54	−2.12	−0.89
*Olea dioica*	0.57	0.23	−0.51	0.48
*Artocarpus heterophyllus*	0.47	2.42	−0.70	1.80
*Syzygium gardneri*	0.29	1.19	0.52	0.91
*Persea macrantha*	−0.13	−0.57	0.92	−1.28
*Actinodaphne malabarica*	−0.19	−3.29	3.02	2.11
*Garcinia indica*	−0.32	1.64	−0.71	0.27
*Elaeocarpus serratus*	−0.68	−0.38	−0.39	−0.74
*Syzygium cumini*	−0.82	1.16	0.66	0.66
*Elaeocarpus tuberculatus*	−1.00	−1.81	−2.11	0.67
*Myristica malabarica*	−1.12	1.93	2.25	−2.39
*Syzygium caryophyllatum*	−1.20	0.69	−0.51	1.24
*Holigarna grahamii*	−3.07	−1.37	−0.69	−1.81

*Note*: Four Principal Component Analysis dimensions with 16 species, explained ~80% variation among species traits.
