# Correction to “Anatomical Traits Explain Drought Response of Seedlings From Wet Tropical Forests”

**DOI:** 10.1002/ece3.74177

**Published:** 2026-09-07

Jhaveri, R. , L. Cannanbilla , K. S. A. Bhat , M. Sankaran , and M. Krishnadas . 2024. “Anatomical Traits Explain Drought Response of Seedlings From Wet Tropical Forests.” Ecology and Evolution 14, no. 9: e70155. 10.1002/ece3.70155.39224158 PMC11366499

Affiliation 2 has been added to the affiliations of the author Meghna Krishnadas. The correct affiliations for Meghna Krishnadas are as follows: “CSIR‐Centre for Cellular and Molecular Biology, Hyderabad, India,” “Academy of Scientific and Innovative Research (AcSIR), Ghaziabad, India,” and “National Centre for Biological Sciences, TIFR, Bangalore, India.”

The online version of this article has been corrected accordingly.

We apologize for this error.

