# Supplementary material for: Anatomical traits explain drought response of seedlings from wet tropical forests
Source: Ecol Evol. 2024 Sep 1;14(9):e70155. doi: 10.1002/ece3.70155 (PMC11366499; doi:10.1002/ece3.70155)
Supplement: Supplementary file 1 — Data S1. [file ECE3-14-e70155-s001.zip › Metadata_Jhaveri etal-drought&traits_10Jan2024.pdf]

File name: **GrowthRates_traits.csv**		
**Column**	**units**	**details**
Sr.		serial number
SPECIES		Species code as given in Appendix table 1
species_code		Seedling unique id
block_code	B1 – B6	Experimental block B1 – B6
treatment		C = Control treatment, D = drought treatment
week_num.x		week of starting experiment, always 1
stem_height.x	cm.	stem height at the start of experiment
week_num.y		week of end of experiment for each seedling
stem_height.y	cm.	stem height at the end of experiment
delta_grth	cm.	stem height difference (stem_height.y–stem_height.x)
grth_rate	cm. week^{-1}	growth rate per week. (delta_grth / week_num.y)
RGR	cm. week^{-1} cm.$^{-1}$	relative growth rate (grth_rate / stem_height.x)
MEAN_SLA	cm.^{2} g.$^{-1}$	mean sla for species
MEAN_LDMC	µm mg^{-1}	mean ldmc for species
MEAN_SS	µm.^{2}	mean stomatal size for species
MEAN_SL	µm.	mean stomatal length for species
MEAN_XD	number µm.$^{-2}$	mean xylem diameter for species
MEAN_SD	number cm.$^{-2}$	mean stomatal density for species
MEAN_VD	cm. cm.$^{-2}$	mean vein density for species
MEAN_RL	mm.	mean root length
MEAN_RA	mm.^{2}	mean root area
MEAN_RD	mm.	mean root diameter
MEAN_SRL	cm. g.$^{-1}$	mean specific root length
MEAN_RF	g. g.$^{-1}$	mean root mass fraction
SI		Seasonality index
SA	cm.^{2} cm.$^{-2}$	Mean Stomatal area fraction (SAF) for species

File name: **Photosynthesis_traits.csv**		
**Column**	**units**	**details**
Sr.		serial number
SPECIES		Species code as given in Appendix table 1
species_code		Seedling unique id
block_code	B1 – B6	Experimental block B1 – B6
treatment		C = Control treatment, D = drought treatment
date		date of photosynthesis reading
n_week		number of week since the start of experiment
photosynthesis_rate	µ mol CO^{2} m^{-2} s^{-1}	photosynthesis rate (As.)
MEAN_SLA	cm.^{2} g.$^{-1}$	mean sla for species
MEAN_LDMC	µm mg^{-1}	mean ldmc for species
MEAN_SS	µm.^{2}	mean stomatal size for species
MEAN_SL	µm.	mean stomatal length for species
MEAN_XD	number µm.$^{-2}$	mean xylem diameter for species
MEAN_SD	number cm.$^{-2}$	mean stomatal density for species
MEAN_VD	cm. cm.$^{-2}$	mean vein density for species
MEAN_RL	mm.	mean root length
MEAN_RA	mm.^{2}	mean root area
MEAN_RD	mm.	mean root diameter
MEAN_SRL	cm. g.$^{-1}$	mean specific root length
MEAN_RF	g. g.$^{-1}$	mean root mass fraction
SI		Seasonality index
SA	cm.^{2} cm.$^{-2}$	Mean Stomatal area fraction (SAF) for species

File name: **Survival_traits.csv**		
**Column**	**unit**	**detail**
Sr.		serial number
SPECIES		Species code as given in Appendix table 1
species_code		Seedling unique id
treatment		C = Control treatment, D = drought treatment
week_num		week of experiment end
DOA	1 = alive, 0 = dead	Survival at the end of experiment
block_code	B1 – B6	Experimental block B1 – B6
MEAN_SLA	$cm.^{2}$ $g.^{-1}$	mean sla for species
MEAN_LDMC	μm mg^{-1}	mean ldmc for species
MEAN_SS	$\mu m.^{2}$	mean stomatal size for species
MEAN_SL	µm.	mean stomatal length for species
MEAN_XD	number $\mu m.^{-2}$	mean xylem diameter for species
MEAN_SD	number $cm.^{-2}$	mean stomatal density for species
MEAN_VD	cm. $cm.^{-2}$	mean vein density for species
MEAN_RL	mm.	mean root length
MEAN_RA	$mm.^{2}$	mean root area
MEAN_RD	mm.	mean root diameter
MEAN_SRL	cm. $g.^{-1}$	mean specific root length
MEAN_RF	g. $g.^{-1}$	mean root mass fraction
SI		Seasonality index
SA	$cm.^{2}$ $cm.^{-2}$	Mean Stomatal area fraction (SAF) for species
